# Institutionalizing Community Health Services in Kenya: A Policy and Practice Journey

**DOI:** 10.9745/GHSP-D-20-00430

**Published:** 2021-03-15

**Authors:** Salim Hussein, Lilian Otiso, Maureen Kimani, Agatha Olago, John Wanyungu, Daniel Kavoo, Rose Njiraini, Sila Kimanzi, Robinson Karuga

**Affiliations:** a Kenya Ministry of Health, Nairobi, Kenya.; b LVCT Health, Nairobi, Kenya.; c Kenya Country Office, United Nations Children's Fund, Nairobi, Kenya.; d U.S. Agency for International Development, Nairobi, Kenya.

## Abstract

The process of institutionalizing community health services in Kenya required strong leadership by the Ministry of Health, effective coordination and support of stakeholders, and alignment of community health with the political priorities at the national and decentralized government levels to facilitate adequate prioritization and financing of the community health strategy.

## Key Messages

- Institutionalizing community health services is a long journey that involves developing relevant policy documents that align with national and global priorities and have the support of political stakeholders.
- A legal framework and continuous engagement with leaders of subnational units is important to ensure standardization, institutionalization, and financing of policies.
- Policy should be informed by evidence generated from within the country to understand what is working well and address challenges that may limit implementation.
- Engage multiple stakeholders to use a multisectoral approach and to harness their various strengths to support policy development.

## INTRODUCTION

Kenya has made tremendous progress in institutionalizing community health services at the policy and practice level. The last 5 years have been particularly instrumental as this period also coincided with key changes globally and in Kenya. Globally, the push for universal health coverage (UHC) since the United Nations declaration of 2012^1^ and the refocus on primary health care (PHC) from the Alma Ata declaration of 1978^2^ and the Astana declaration of 2018^3^ has been instrumental in informing Kenya's more recent policy priorities as the country signed onto them.

In 2017, the Kenyan President made UHC a priority as part of the Big Four Agenda for development^4^^,^^5^ to ensure all Kenyans could access the services they required without experiencing financial hardship. This progress would not have been possible without strong government leadership and a strong partnership and engagement with devolved subnational governments, referred to as counties and other stakeholders.

In Kenya, community health volunteers (CHVs) are key for delivery of PHC services and UHC. This cadre of lay health workers gained prominence after the Alma Ata Declaration of 1978.^2^ In response to declining health indicators from the 1990s, Kenya's Ministry of Health (MOH) first launched the Community Health Strategic Plan in 2006 (Figure).^6^ The strategic plan focused on providing community level health services for all, building the capacity of the community health extension workers (CHEWs), strengthening health facility-community linkages, and strengthening the community to progressively realize their rights for accessible and quality care.

**FIGURE:**
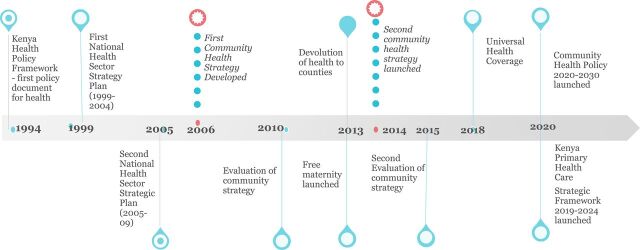
Evolution of the Community Health Strategy in Kenya

In 2014, the MOH operationalized the second national health sector strategic plan (2014–2019),^7^ which aimed to further reverse declining health indicator trends. This revised strategy focused on improving the delivery of integrated, comprehensive, and quality community health services for all population cohorts; strengthening data demand and information use at all levels; and bolstering mechanisms for resource mobilization and management for sustainable implementation of community health services. Community units—identified as the basic geographical unit for delivery of basic health services (health prevention, promotion and education, targeted disease prevention, and basic curative services)—comprised 5,000 people or 1,000 households. Each community unit was served by CHVs that were supervised by CHEWs.^7^ The second strategy also addressed gaps in attrition of CHVs and recognizing the heterogeneity of the country when implementing the strategy.

Despite having a revised strategy, the implementation of full community health services at the county level was hampered by lack of a community health policy that served as a legal framework.^8^

This article is based on reflections of the policy makers (leadership of the Division of Community Health in the MOH), donors, and implementing partners who were involved in the processes that have led to institutionalizing the community health services. We describe how development of the community health policy has contributed to institutionalization of community health services and increased the visibility of the community strategy as a key pillar toward achieving the UHC and PHC priorities in Kenya.

## THE COMMUNITY HEALTH POLICY

In July 2020, Kenya launched its first Community Health Policy 2020–2030^8^ alongside the Primary Health Care Strategic Framework 2019–2024^9^ at an event led by the Cabinet Secretary for Health in recognition of the key role that CHVs play in delivering PHC services.

The policy's key objectives are to provide guidance for establishing and implementing a strong, comprehensive, integrated, equitable, holistic, and sustainable community health structure in Kenya (Table). The policy provides the legal framework to facilitate implementation and achievement of 100% coverage with community units and recognition of community health personnel by the counties. This policy addresses issues such as recruitment, remuneration, training, and deployment of the community health workforce and a stronger community health information system.

**TABLE.** Summary of the Community Health Policy Objectives, Kenya^6^

**General Objective**	To provide policy guidance for the establishment and implementation of a strong, equitable, holistic, and sustainable community health structure
**Specific Policy Objectives**	
**1. Leadership and Governance**	Secure effective leadership and governance in the formation, maintenance, and management of community health structures and participation mechanisms
**2. Community Health Workforce**	Ensure the recruitment and retention of community health human resources for health, including obtaining appropriate numbers and strengthening mechanisms for capacity building and supportive supervision of community health personnel
**3. Service Delivery**	As per the community health strategy, ensure provision of high-quality community health services at the household and community level, including referral and follow-up services
**4. Community-Based Health Information System**	Support the development and strengthening of community-based health information system and the monitoring and evaluation of systems to sufficiently inform the implementation of community services at all levels
**5. Health Products and Technologies**	Promote and strengthen supply chain systems for community health that are integrated into the government-led reporting systems and that link facilities including the use of available technology
**6. Financing for Community Health**	Provide various mechanisms for mobilizing, managing, and appropriately allocating resources for sustainable financing and delivery of community health services at all levels
**7. Monitoring, Evaluation, Research and Community-Based Surveillance**	Provide for community health services and human resources data and knowledge management that will inform evidence-driven decision making

### Key Factors That Contributed to the Successful Establishment of the Policy

We identified 4 factors that had the most significance in ensuring successful policy development and ensuring progress in institutionalizing community health services. These factors were the result of reflection and iteration of various factors that arose during the writing of the article.

#### 1. The Importance of Context (Devolution/Decentralization)

In 2013, Kenya devolved health and other services from the central government to 47 new subnational governments known as counties, which are semiautonomous units responsible for implementation of health services. The national government is responsible for training and development of policies and guidelines. Devolution presented a number of challenges,^10^^,^^11^ including the differences in how counties implemented the second 2014–2019 community strategy. Some county leaders did not recognize the CHVs' role in the health system. Other counties developed their own models of the strategy and overhauled the entire program with varying levels of success.^12^ Some counties served as model counties by establishing mechanisms for remunerating CHVs (Siaya County), and others adapted the strategy to prioritize their health needs.^13^ The varying implementation of the community strategy highlighted to the national MOH the urgent need for development of a national community health policy in response to the changes to the legal policy and institutional framework governing the health sector. This policy would be informed by county priorities and would enable buy-in and implementation by counties.

The varying implementation of the community strategy and changes to the legal policy framework highlighted the need for a national community health policy.

#### 2. Evidence-based Policy Making

Community health policy and guidelines development in Kenya has been informed by research and evaluations conducted by the MOH in partnership with various stakeholders. The first strategy (2006) was informed by implementation research that demonstrated improved health indicators due to work performed by CHVs. After the first 5 years of scale-up, a countrywide evaluation revealed that the strategy was successful in improving indicators such as hygiene, sanitation, uptake of antenatal care services, and child health (immunization and diarrhea). The evaluation also identified high CHV attrition due to lack of a reward or remuneration system, inadequate empowerment of community members, and weak accountability and governance structures.^14^

After this evaluation, the 2014–2019 community strategy was defined, recognizing the challenge of nonremunerated CHVs and proposing a monthly stipend equivalent to US$20. It proposed a new structure for the community unit with fewer CHVs and 5 CHEWs who could provide health promotion and basic curative services at the household level. The revised strategy also recognized the need for adaptation of the strategy based on the diverse socioeconomic and ecological contexts seen in the country (i.e., urban, agrarian, nomadic, and pastoralist communities).^7^ In 2015, various stakeholders in all counties conducted a national evaluation that provided the evidence demonstrating the need for a community health policy, due to the lack of a legislative framework, that counties could use to advocate for and budget for CHV remuneration and other CHS costs.^15^

A 2018 national assessment of the community health strategy in the 47 counties (unpublished) found that most counties did not have specific community health policies or guidelines, and they relied on the national guidelines and the constitution to guide their community health planning. About 10 counties had a community health bill (legislation to recognize CHVs in law). About two-thirds of the counties reported allocating funds for community health, albeit inadequate. A third of the counties reported providing financial incentives to CHVs although this was inconsistent. The assessment identified challenges related to the varied implementation of community strategy across counties: varying number of CHVs per community unit, training, and supervision structures; lack of budgetary allocation and proper utilization of funds for community health in most counties with reliance on donors or nongovernmental organizations (NGOs) in others; low quality of community health services due to lack of training, commodities, supervision, and quality improvement of CHWs; and poor coordination of partners/stakeholders including funders at the county level. Finally, lack of a legal framework to sustain the funding for community health services at the national or county level was a common and urgent problem similar to that identified in the 2015 evaluation and informed the need to expedite finalization of the community health policy that had been in development since 2016.

#### 3. Stakeholders' Engagement

The policy's development and launch was a result of concerted efforts since 2016 by many stakeholders, including the county governments and partners led by the MOH. The community health program has benefited from support from various stakeholders at the national and subnational levels including development partners such as United Nations Children's Fund (UNICEF), U.S. Agency for International Development (USAID), NGOs, religious bodies, community-based organizations, and community leaders. This has been in line with recommendations from various global guidelines.^16^^,^^17^

In 2017, Kenya's MOH officials and stakeholders, including UNICEF, USAID, and NGO representatives that are members of the Community Health Technical Working Group, attended the first Institutionalizing Community Health Conference in Johannesburg, South Africa. This delegation developed an action plan toward institutionalizing community health in Kenya, reflecting on progress made, key challenges, and priorities for achieving goals based on their knowledge of the Kenyan community health landscape.

The stakeholders set 3 priority objectives that have contributed to the institutionalization of community health described in this article.

1. Finalize the Community Health Services Policy: The progress had begun in 2016 but had stalled and needed engagement with counties for input and finalization.
2. Conduct an assessment/evaluation of Kenya's community health services: Although the revised community strategy had been launched in 2014, it had been developed prior to devolution and was due for a review that would meet the varying needs of counties. This process informed an evaluation in 2018 that is being used to revise the strategy that is ongoing at the time of submission of this article.
3. Increase the visibility of community health services: Stakeholders felt that community health services in Kenya were not prioritized beyond the Division of Community Health Services or adequately financed by the MOH and other donors^14^ despite being in existence and mentioned in several government policy documents since 2006. At the time of the conference, the MOH Community Health and Development Unit only received salaries from the government and completely relied on donors and NGOs to conduct operational activities in their work plans. In addition, although the community strategy had been in place since 2006, it was not recognized, integrated, or budgeted for across all program areas and priority government initiatives, including *Linda Mama* (free maternal health care) and UHC programs that had been rolled out by the government. The stakeholders agreed to utilize all opportunities to advocate for CHWs in all programs.

The action plan helped to synergize and catalyze efforts toward strengthening and institutionalizing community health services in Kenya by all stakeholders. The MOH Division of Community Health Services regularly convened stakeholders through technical working groups to conduct a national evaluation of the community strategy and support the development of the policy and other relevant policy documents and guidelines. Throughout all these processes, county governments were engaged to provide input into the development as they are responsible for the implementation of the community health strategy in the county. The result has been national documents that have input from a wide range of stakeholders and are likely to be sustained.

The action plan helped to synergize and catalyze efforts toward strengthening and institutionalizing community health services in Kenya by all stakeholders.

#### 4. Political Leadership and Alignment with Political Priorities

The role of politics in informing health policies and financing cannot be understated.^18^ The stakeholders involved in developing the community health policy recognized that for community health to be integrated and institutionalized in the health system in Kenya, there had to be buy-in from the top government leadership. They identified opportunities or policy windows^19^ that had presented themselves to support recognition of the community strategy. The most important was the UHC agenda.

As part of the Kenya President's Big Four Agenda for development 2017–2022, the UHC agenda aims to ensure affordable and quality health care for all and specifically mentions scaling up CHVs as a priority initiative. The Cabinet Secretary for Health attended the Global Conference on Primary Health Care in Astana, Kazakhstan, in October 2018, and Kenya endorsed the declaration on PHC that prioritized working with CHWs.^20^ The Primary Health Care Strategy 2019–2024, which incorporates CHVs as members of the multidisciplinary team at the PHC level, is set to drive the UHC agenda in Kenya.^9^ The Cabinet Secretary for Health has committed to prioritize institutionalization and integration of community health in all health policies.

The 47 county governments have all endorsed the UHC agenda, the Primary Health Care Strategy, and the Community Health Policy as a result of the political buy-in from the President and the Cabinet Secretary for Health. This has resulted in increased commitments from counties to prioritize and budget for CHVs and other community health cadres in their counties.

### Milestones in the Journey to Institutionalizing Community Health Services

The consultative process of developing the Community Health Policy began in 2016 and was completed in 2020 following several consultative meetings with the county governments. Other policy level events happened in tandem, which strengthened the community health system and advocacy. First, finalization of the National Kenya Health Act^21^ institutionalized the community level as tier 1 in the health system. Second, publication of the Investment Case for Community Health Services demonstrated a 9.4 to 1 return on investment when Kenya invests in community health services.^22^ This was similar to global investment cases that showed that investing in community health can generate up to 10 times return on investment and was key to supporting advocacy efforts to finance community health at the national and county government level.^23^ To ensure quality of CHW programs, the USAID SQALE project developed a mechanism for implementing quality improvement at the community level by adapting the Kenya Quality Model for Health.^24^

Some of the key milestones to date that resulted from the processes undertaken by the MOH and its stakeholders to institutionalize community health in the health system are described here.

1. The CHVs have played a significant role in the coronavirus disease (COVID-19) pandemic response in Kenya.^25^ With training and support from the MOH and stakeholders, they have supported infection prevention control, contact tracing, and home-based care. This has gained them recognition and special mention by the Cabinet Secretary for Health, county governors, and the President on many occasions. We anticipate that this will cement the financing of the CHW programs and further institutionalization.
  With training and support from the MOH, CHVs played a significant role in the COVID-19 pandemic response in Kenya.
2. In line with the community health strategy, the government has disbursed funds to train 31,780 CHVs to increase coverage of community units to 100% nationally. The government has also committed to train and recruit 2,000 salaried CHEWs through government-owned Kenya medical training colleges across the country.
3. The MOH is working with the Senate on the Community Health Services Bill,^26^ which will anchor the community health services and formally recognize tier 1 services, giving it a legal mandate to be funded by counties. This will augment the existing county-level community health service bills that have given CHVs recognition and will allow them to earn a stipend in some counties.
4. There have been advancements in developing systems and strategies to strengthen community health services. The following are ongoing processes at the time of publication of this article: development of a revised community strategy (2020–2025) that incorporates findings from the national evaluation; development of community-based surveillance system to enhance early identification of outbreaks at community level; and digitization of the community-based health information system–electronic CHIS (E-CHIS).

### Challenges in Institutionalization of Community Health Services

Despite the gains made in institutionalizing community health in Kenya, a lot of work still needs to be done by the MOH and stakeholders to sustain these gains. Some of the priority activities are listed here.

- Sustained advocacy for funding for community health services from domestic sources (national and county level) by MOH staff, NGOs, and development partners.^27^ This will supplement the financial support received from bilateral and multilateral funders.
- Completion of the legislation processes to ensure community health services are delivered and financed through legitimate and sustainable policy frameworks.
- Strengthening of accountability mechanisms at different government and health system levels starting with communities to demand quality and sustained primary and universal health care. ^28^
- Ensuring quality of community health programs by aligning them to the policy, standardized training, commodities, supervision, and quality improvement mechanisms as described in the World Health Organization guidelines on community health.^29^

## CONCLUSION

Kenya's MOH has made significant progress in institutionalizing community health services in policy. The lessons learned on understanding context, use of evidence, meaningful engagement of stakeholders, and alignment to political priorities are important to inform successful policy engagement processes for others in a similar situation. Sustaining these gains will be a key priority for the technocrats in the MOH and the stakeholders to ensure CHW programs remain a political priority that is financed as a core component of the health system.

## Notes

**Peer Reviewed**
